# Cardiopulmonary Imaging Utilization and Findings among Hospitalized COVID-19 Patients in Latin America (From “RIMAC: Registry IMAging Cardiopulmonary among Hospitalized COVID-19 Patients in LATAM”)

**DOI:** 10.5334/gh.1134

**Published:** 2022-08-01

**Authors:** Salvador V. Spina, Marcelo L. Campos Vieira, César J. Herrera, Ana G. Munera Echeverri, Pamela Rojo, Alma S. Arrioja Salazar, Zuilma Y. Vázquez Ortiz, Roberto Baltodano Arellano, Graciela Reyes, Rocío Aceves Millán, Juan A. Calderón González, Ana C. Camarozano, Edgar Avilés, Marco A. Cabrera, María F. Grande Ratti, Jorge Lowenstein, Rodrigo Hernández Vyhmeister, Pamela Piña Santana, Jaime A. Ibarra Burgos, Alejandra Rivera, Beatriz A. Fernández Campos, Kelly M. Cupe Chacalcaje, Mariela De Santos, Tania R. Afonso, Tomás Miranda Aquino, Ana L. Lalyre Acosta, Beatriz Domínguez, Federico Campos, Sergio M. Alday Ramírez, Angela V. Cachicatari Beltran, Daniela Alvarez, Patricia de Oliveira Roveri, Carlos Rosales Ixcamparij, Ender Otoniel González, Pedro Vargas, Maximiliano Flores Flamand, Rosa L. López Martínez, Luciana Meza, Samira Saady Morthy, Rudy Ovalle, Stalin Martínez, Oscar A. Pérez Orpinel, Mauricio Potito, María Verónica Espinoza Arregui, Jorge Marte Baez, Consuelo Orihuela Sandoval, Marcos Granillo Fernandez, Rohit Loomba, Saúl Flores, José M. Hernández, Ricardo Pignatelli

**Affiliations:** 1 Hospital Aeronáutico Central, Buenos Aires, AR; 2 Hospital Israelita Albert Einstein, São Paulo, BR; 3 CEDIMAT, Santo Domingo, DO; 4 Hospital General de Medellín, Medellín, CO; 5 Clínica Dávila, Santiago de Chile, CL; 6 Instituto Nacional de Ciencias Médicas y Nutrición “Salvador Zubirán,” Ciudad de México, MX; 7 HNG Almenara Irigoyen, Lima, PE; 8 Hospital El Cruce, Provincia Buenos Aires, AR; 9 Centro Médico Nacional 20 de Noviembre, Ciudad de México, MX; 10 Hospital General de Zona Número 4, Monterrey, MX; 11 Hospital Nossa Senhora das Graças y Universidade Federal do Paraná, BR; 12 Complejo Hospitalario Dr. Arnulfo Arias Madrid, Ciudad de Panamá, PA; 13 TECNISCAN Hospitalia, Ciudad de Guatemala, GT; 14 Internal Medicine Research Area, Hospital Italiano de Buenos Aires, AR; 15 Instituto de Investigaciones Médicas, Buenos Aires, AR; 16 Hospital de la Fuerza Aerea, Santiago de Chile, CL; 17 Medicina Interna Universidad CES, Medellín, CO; 18 Technique, Hospital El Cruce, Provincia Buenos Aires, AR; 19 Hospital Israelita Albert Einstein, São Pablo, BR; 20 Technique, TECNISCAN Hospitalia, Ciudad de Guatemala, GT; 21 Advocate Children’s Hospital/Rosalind Franklin University of Medicine and Science, Chicago, IL, US; 22 Texas Children’s Hospital/Baylor School of Medicine, Houston, TX, US; 23 Cardiolink Estudios Cardiovasculares, Monterrey, MX; 24 Children’s Hospital, Baylor College of Medicine, Houston, US

**Keywords:** Cardiopulmonary Images, SARS-CoV-2, COVID-19, LATAM, RIMAC

## Abstract

**Objectives:**

Describe the use and findings of cardiopulmonary imaging—chest X-ray (cX-ray), echocardiography (cEcho), chest CT (cCT), lung ultrasound (LUS), and/or cardiac magnetic resonance imaging (cMRI)—in COVID-19 hospitalizations in Latin America (LATAM).

**Background:**

There is a lack of information on the images used and their findings during the SARS-CoV-2 pandemic in LATAM.

**Methods:**

Multicenter, prospective, observational study of COVID-19 inpatients, conducted from March to December 2020, from 12 high-complexity centers, in nine LATAM countries. Adults (>18 years) with at least one imaging modality performed, followed from admission until discharge and/or in-hospital death, were included.

**Results:**

We studied 1,435 hospitalized patients (64% males) with a median age of 58 years classified into three regions: Mexico (Mx), 262; Central America and Caribbean (CAC), 428; and South America (SAm), 745. More frequent comorbidities were overweight/obesity, hypertension, and diabetes. During hospitalization, 58% were admitted to the ICU. The in-hospital mortality was 28%, and it was highest in Mx (37%).

The most frequent images performed were cCT (61%), mostly in Mx and SAm, and cX-ray (46%), significant in CAC. The cEcho was carried out in 18%, similarly among regions, and LUS was carried out in 7%, with a higher frequently in Mx. Abnormal findings on the cX-ray were peripheral or basal infiltrates, and in cCT abnormal findings were the ground glass infiltrates, more commonly in Mx. In LUS, interstitial syndrome was the most abnormal finding, predominantly in Mx and CAC.

Renal failure was the most prevalent complication (20%), predominant in Mx and SAm. Heart failure developed in 13%, predominant in Mx and CAC. Lung thromboembolism was higher in Mx while myocardial infarction was in CAC.

Logistic regression showed associations of abnormal imaging findings and their severity, with comorbidities, complications, and evolution.

**Conclusions:**

The use and findings of cardiopulmonary imaging in LATAM varied between regions and had a great impact on diagnosis and prognosis.

## Introduction

### Background/rationale

The COVID-19 pandemic is one of the largest and most active threats to health care in living memory. As the impact of the virus continues, systems of care around the world have responded with unprecedented protective measures.

SARS-CoV-2 predominantly affects adults, and disease severity increases with age and number of comorbidities. COVID-19 infection is mainly characterized by upper airway inflammation, which can progress into interstitial pneumonia and eventually to acute respiratory distress syndrome (ARDS) in the most severe cases. Cardiovascular complications such as thromboembolic phenomena, acute coronary syndrome, heart failure, and renal dysfunction are known to occur [1,2,3,4].

Cardiopulmonary imaging plays an essential role in the diagnosis of SARS-CoV-2 infection and its complications. Imaging can assess the extent of disease, prognosis, and evaluation of therapeutic interventions [1,2,3,4,5,6,7]. Imaging services resources (ISR) such as electrocardiogram (ECG) [8,9], chest X-ray (cX-ray) [10], echocardiogram (cEcho) [11,12,13,14], lung ultrasound (LUS) [15,16], and chest computed tomography (cCT) [17,18] have been at the front line of the pandemic. Each technique offers well-known advantages, however despite the high number of cases of infection and deaths from COVID-19, their specific application and utilization in low- and middle-income countries remains unknown.

Preventive distancing and biosecurity measures during testing can protect patients and staff, thus abbreviated evaluations and imaging interventions have become the norm.

### Objectives

The main objectives were as follows:

1. To describe the use and findings of cardiopulmonary imaging modalities performed for the diagnosis and treatment of patients hospitalized for SARS-CoV-2 infection in Latin America (LATAM)
2. To compare the differences between three geographic regions of LATAM countries: Mexico (Mx), Central America and Caribbean (CAC), and South America (SAm)

We also describe their demographic parameters, comorbidities, in-hospital events (or clinical complications), laboratory results, and concomitant treatments of all included patients.

## Methods

### Study design and setting

We conducted a multicenter, prospective, observational study based on the RIMAC registry that included adult patients with SARS-CoV-2 disease admitted from March to December 2020, in 12 high-complexity centers (level III or IV) from nine countries—Argentina, Brazil, Colombia, Chile, Guatemala, Mexico, Panama, Perú, and the Dominican Republic—which were divided into three geographic regions. All the centers had availability of the total imaging modalities analyzed. The total number of referral beds in each center was greater than 185, which doubled and tripled according to the needs of each region. We used STROBE for observational studies as a reporting guideline.

### Participants

The cohort included a consecutive sample of patients, followed up from admission until discharge and/or in-hospital death. Inclusion criteria were patients >18 years old, positive COVID-19 CRP and/or COVID-19 positive IgM and IgG antibodies, hospitalized status, and at least one imaging modality performed according to each treating physician’s criteria (cX-ray, cEcho, LUS, cCT, or cMRI).

The exclusion criteria were patients <18 years old, lack of complete documentation on COVID-19 infection, nonhospitalized patients, failure to perform an imaging modality, or inadequate quality for diagnosis and/or treatment (cX-ray, cEcho, LUS, cCT, or cMRI).

### Data source proceeding/variables

The baseline data (demographic, epidemiological, clinical, imaging, laboratory, treatment, and outcome) was reviewed and collected by the research team, came from the medical records of the patients, using a standardized unique form (RIMAC Registry) in all centers, and was securely stored in a database created for this purpose for subsequent analysis. Each patient was assigned a de-identifying code. Local IRB approval from each center was obtained, and researchers were not involved in direct care of the subjects. Informed consent was not obtained in all centers because it was an observational, noninterventional study of registry research with minimal risk. Most patients were aware that their data was going to be used for research purposes through verbal explanations.

Patients were prospectively monitored for major complications during hospitalization.

### Statistical analyses

All statistical analyses were conducted using STATA, version 17.0. Descriptive variables were summarized using proportions for categorical variables, and mean (± standard deviation) or median (interquartile range) as appropriate.

We divided the cohort into three geographic regions for multiple comparisons. These were identified and agreed upon by the authors a priori: Mexico (Mx) as a representation of the Latin population of North America; Central America, and Caribbean (CAC), which included Guatemala, Panamá, and the Dominican Republic; and South America (SAm), which included Argentina, Brazil, Chile, Colombia, and Perú.

Comparisons between regions were performed using Pearson’s χ2 test with Bonferroni’s correction for categorical variables. Meanwhile, for continuous variables we used analysis of variance (ANOVA) or Kruskal-Wallis H test to compare differences among groups. In these cases of multiple comparison, a p-value of ≤0.016 was considered strength of evidence.

In addition, univariate and multivariate logistic regressions were performed to explore factors associated with a specific clinical imaging modality. The modality of choice was based on the treating physician, the clinical presentation, the availability of diagnostic images, and local guidelines in place for imaging. We have selected clinical variables as potential confounders of adjustment. The adjusted analysis was based on the comparison from different regions: Mx, CAC, and SAm were defined as the exposure variable. The strength of evidence level was set at 0.05 (two-tailed), and odds ratios (OR) and their respective 95% confidence intervals (95%CI) were reported.

## Results

### Participants/descriptive data

**Figure 1:**
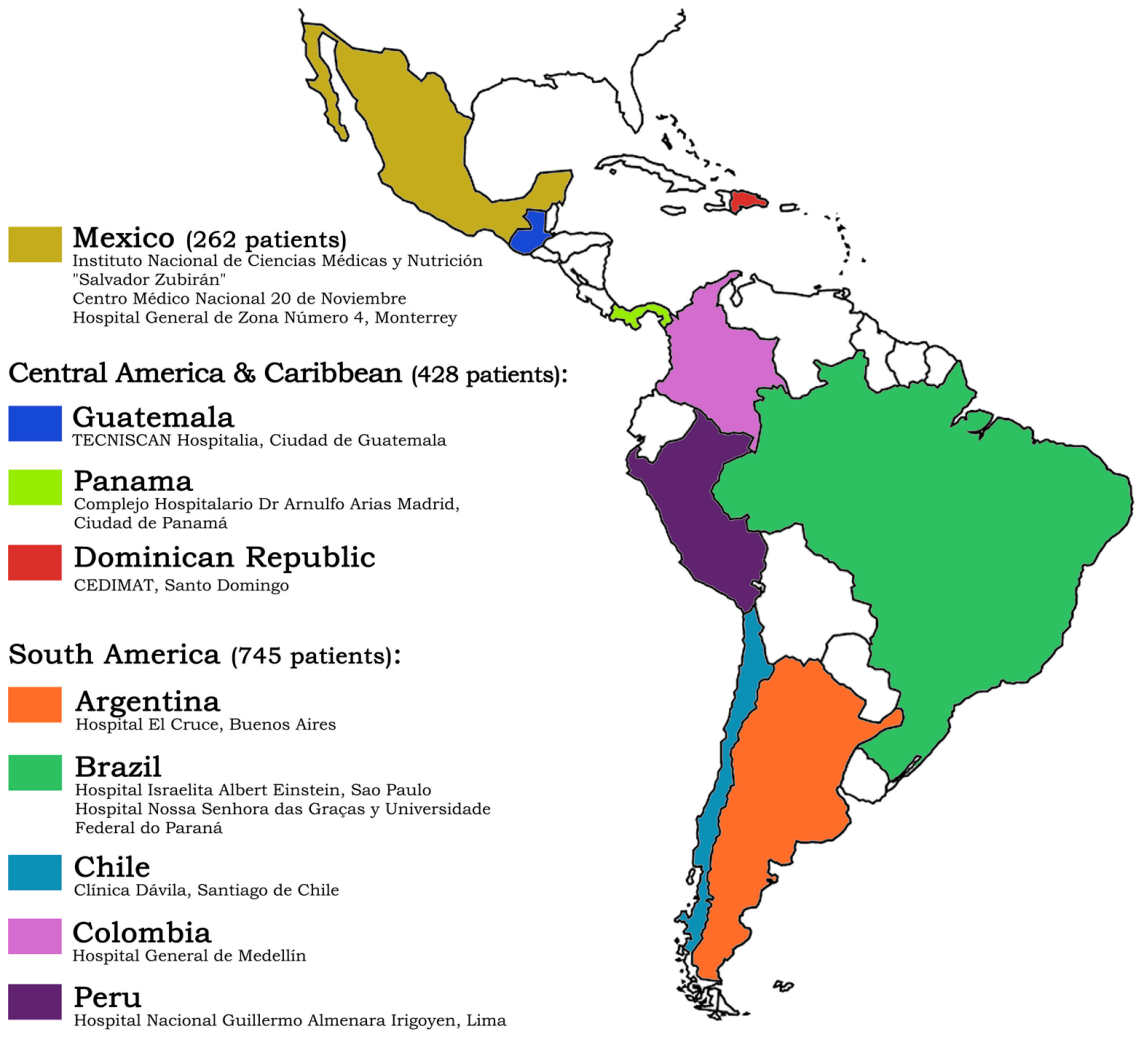
RIMAC Registry. Participating regions, countries and institutions (1,435 patients).

There were 1,549 hospitalized patients recorded in the RIMAC registry, and 114 patients were excluded due to a lack of complete documentation on COVID-19 infection. The remaining 1,435 patients were included in the study (64% males) with a median age of 58 (SD 16.6); their geographical distribution was 262 from Mx, 428 from CAC, and 745 from SAm (Figure 1).

A total of 708 patients (49%) were admitted to the ward, and 727 (51%) patients were initially admitted to the ICU. We found strong evidence that patients were more frequently admitted in wards in Mx in comparison with the other regions. The ICU patients subsequently increased to 836 (58%) during total hospital stay (most of them were Mexican patients) and thus normalized the differences among regions. The median number of hospitalization days was 17 (SD 17.2), and the median ICU days was 13 (SD 14.2). This was significantly lower in CAC.

The most common comorbidities were overweight/obesity (61%), hypertension (45%), and diabetes (27%). There were differences across regions: body mass index (BMI) was registered in 1,180 patients, and overweight/obesity (BMI ≥ 25) was significantly more prevalent in Mexico (42%) especially due to obesity (BMI ≥ 30). Hypertension (HBP) was significantly higher in CAC (60%), and diabetes (DBT) predominated in Mx and CAC (34%). Ischemic heart disease, chronic obstructive pulmonary disease (COPD), renal failure, and myocardiopathy were below 7% of the population (Table 1).

**Table 1:** Demographic variables and baseline comorbidities.


	GLOBAL (*n* = 1,435)	MEXICO (MX) (*n* = 262)	CENTRAL AMERICA AND CARIBBEAN (CAC) (*n* = 428)	SOUTH AMERICA (SAM) (*n* = 745)	p-VALUE*

***Demographic Variables***					

Age (years)	57.95 (SD 16.62)	55.56 (SD 15.41)	59.86 (SD 16.91)	57.69 (SD 16.77)	0.002 0.003 0.094 0.222

Male gender	64.46% (925)	58.40% (153)	70.33% (301)	63.22% (471)	0.004 0.001 0.014 0.166

***Baseline Comorbidities***					

Overweight/obesity (BMI ≥ 25)	61.25% (879)	80.92% (212)	64.95% (278)	52.21% (389)	0.001 0.001 0.001 0.001

Overweight (BMI 25–29)	35.75% (513)	38.55% (101)	43.93% (188)	30.07% (224)	0.001 0.165 0.001 0.012

Obesity(BMI ≥ 30)	25.51% (366)	42.37% (111)	21.03% (90)	22.15% (165)	0.001 0.001 0.654 0.001

HBP	45.37% (651)	36.26% (95)	60.28% (258)	40.00% (298)	0.001 0.001 0.001 0.286

DBT	26.83% (385)	34.35% (90)	33.64% (144)	20.27% (151)	0.001 0.849 0.001 0.001

Former tobacco use	14.15% (203)	6.87% (18)	19.63% (84)	13.56% (101)	0.001 0.001 0.006 0.004

Active tobacco use	8.43% (121)	13.74% (36)	7.94% (34)	6.85% (51)	0.002 0.014 0.485 0.001

Ischemic heart disease	6.76% (97)	4.96% (13)	9.58% (41)	5.77% (43)	0.019 0.028 0.015 0.623

COPD	6.62% (95)	2.67% (7)	2.10% (9)	10.60% (79)	0.001 0.630 0.001 0.001

Renal failure	4.32% (62)	1.91% (5)	2.80% (12)	6.04% (45)	0.003 0.462 0.013 0.008

Myocardiopathy	4.74% (68)	0.00% (0)	9.11% (39)	3.89% (29)	0.001 0.001 0.001 0.001

Valvular heart disease	1.74% (25)	2.29% (6)	2.57% (11)	1.07% (8)	0.128 N/A

HIV	0.42% (6)	0.38% (1)	0.00% (0)	0.67% (5)	0.229 N/A


*Notes*: * Each cell contains four p-values. The first p-value corresponds to chi-squared test (for categorical variables) or one-way ANOVA test (for numerical variables). Only a *p* ≤ 0.016 was considered statistically significance. The second, third, and fourth p-values correspond to multiple comparisons between groups: Mx and CAC, CAC and SAm, and SAm and Mx, respectively. We used multiple chi-squared tests and ANOVA with Bonferroni correction. HBP: high blood pressure; DBT: diabetes; COPD: chronic obstructive pulmonary disease; HIV: human immunodeficiency virus.

### Main results

#### Cardiopulmonary imaging utilization

The most common cardiopulmonary imaging performed in our cohort were cCT (61%) and cX-ray (46%); cEcho was carried out in 18% of patients, and LUS was carried out in 7% of patients. The use of imaging modalities was different across regions: cCT was more frequent in Mx and SAm, and cX-ray was significantly more common in CAC. cEcho was almost the same among regions with a small predominance in Mx. LUS was significantly more common in Mx. Brazil had the highest use of both modalities if we analyze based on country. ECG use was limited (24%) due to the biosafety measures implemented during COVID-19. The lowest use was in SAm (except Brazil) (Table 2). cMRI was performed in only one patient in the cohort and was not taken into account for the statistical analysis.

**Table 2:** Image modalities used.


	GLOBAL (*n* = 1,435)	MEXICO (MX) (*n* = 262)	CENTRAL AMERICA AND CARIBBEAN (CAC) (*n* = 428)	SOUTH AMERICA (SAM) (*n* = 745)	p-VALUE*

**Chest computed tomography (cCT)**	61.53% (883)	69.08% (181)	32.24% (138)	75.70% (564)	0.001 0.001 0.001 0.036

**Chest X-ray (cX-ray)**	45.99% (660)	38.55% (101)	91.12% (390)	22.68% (169)	0.001 0.001 0.001 0.001

**Echocardiogram (cEcho)**	18.54% (266)	24.43% (64)	18.93% (81)	16.24% (121)	0.013 0.085 0.241 0.003

**Lung ultrasound (LUS)**	7.25% (104)	22.14% (58)	0.23% (1)	6.04% (45)	0.001 0.001 0.001 0.001

**Electrocardiogram (ECG)**	24.32% (349)	41.22% (108)	37.62% (161)	10.74% (80)	0.001 0.346 0.001 0.001


*Notes*: * Each cell contains four p-values. The first p-value corresponds to chi-squared test (for categorical variables) or one-way ANOVA test (for numerical variables). Only a *p* ≤ 0.016 was considered statistically significance. The second, third, and fourth corresponds to multiple comparisons between groups: Mx and CAC, CAC and SAm, and SAm and Mx, respectively. We used multiple chi-squared tests and ANOVA with Bonferroni correction.

A multivariate analysis of the use of images was carried out considering confounding factors such as age, sex, hypertension, overweight/obesity, diabetes, renal failure, heart failure, hospital stay, ICU stay, mechanical ventilation, and death. The calculated adjusted ORs show that there were no differences among the regions, and they do not appear to add significant variation or impact.

### Findings

**Figure 2:**
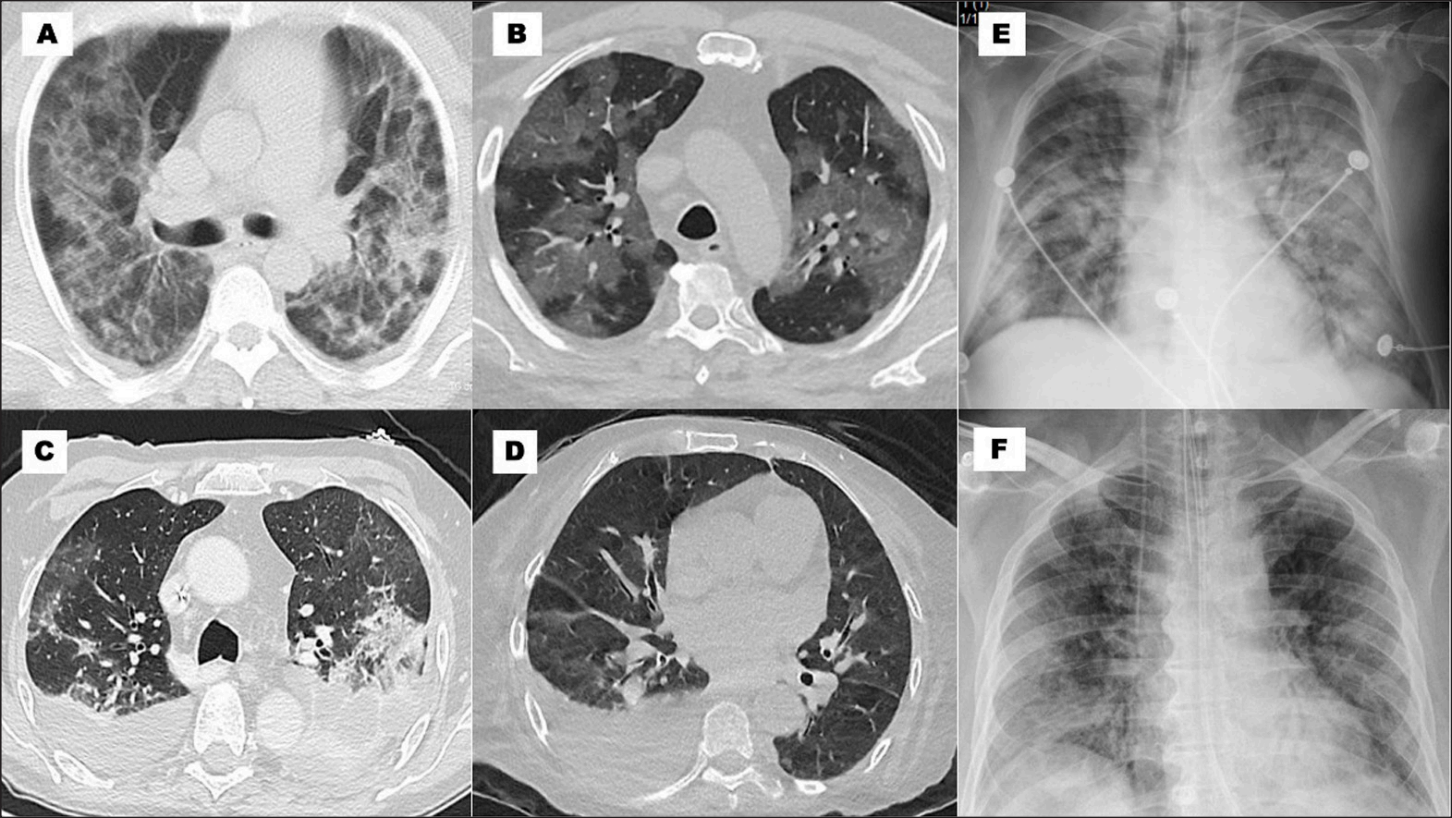
Image patterns. **(A)** cTC: infiltrates in ground glass >50%, crazy paving and alveolar consolidation bilateral; **(B)** cTC: infiltrates in ground glass >50% bilateral; **(C)** cTC: infiltrates in ground glass 25–50% and pleural effusion bilateral; **(D)** cTC: infiltrates in ground glass <25% and pleural effusion unilateral; € cX-ray: peripheral, basal, and hilar infiltrates; ground glass pattern; and consolidation; (F) cX-ray: basal and peripheral infiltrates, and ground glass pattern.

The imaging findings and their regional distribution are shown in Table 3. The most frequent patterns of the cX-ray were peripheral, basal, and ground glass infiltrates with a significant prevalence of abnormalities in Mx. The ground glass appearance of peripheral or subpleural infiltrates on cCT was found in 89% of cases; the most severe subtypes (infiltrates >50%) were significantly higher in Mx (Figure 2). The left ventricular ejection fraction (LVEF) was calculated in all cEcho performed (266 patients) with a mean value of 57% being almost the same in the three regions.

**Table 3:** Image patterns, global and regional.


	GLOBAL (*n* = 1,435)	MEXICO (MX) (*n* = 262)	CENTRAL AMERICA AND CARIBBEAN (CAC) (*n* = 428)	SOUTH AMERICA (SAM) (*n* = 745)	p-VALUE*

**Chest computed tomography (cCT)**	61.53% (883)	69.08% (181)	32.24% (138)	75.70% (564)	0.001 0.001 0.001 0.036

Infiltrates in ground glass	89.35% (789/883)	99.45% (180/181)	87.68% (121/138)	86.52% (488/564)	0.001 0.001 0.719 0.001
_______*% of pulmonary involvement* * <25%* * 25–50%* * >50%*	21,29%(168/789) 32.95% (260/789) 45.75% (361/789)	13.33% (24/180) 18.33% (33/180) 68.33% (123/180)	33.06% (40/121) 41.32% (50/121) 25.62% (31/121)	21.31% (104/488) 36.27% (177/488) 42.42% (207/488)	0.001 N/A

Crazy Paving	30.01%(618/883)	20.99% (38/181)	45.65% (63/138)	29.08% (164/564)	0.001 0.001 0.001 0.033

Alveolar Consolidation	36.58% (323/883)	35.36% (64/181)	42.75% (59/138)	35.46% (200/564)	0.261 N/A

Pleural Effusion	10.76% (95/883)	12.71% (23/181)	18.84% (26/138)	8.16% (46/564)	0.001 0.132 0.001 0.066

**Chest X-Ray (cX-ray)**	45.99% (660)	38.55% (101)	91.12% (390)	22.68% (169)	0.001 0.001 0.001 0.001

Basal infiltrates	51.97% (343/660)	83.17% (84/101)	41.28% (161/390)	57.99% (98/169)	0.001 0.001 0.001 0.001

Peripheral infiltrates	63.33% (418/660)	89.11% (90/101)	58.97% (230/390)	57.99% (98/169)	0.001 0.001 0.828 0.001

Hilar infiltrates	39.24% (259/660)	54.46% (55/101)	44.87% (175/390)	17.16% (29/169)	0.001 0.085 0.001 0.001

Ground glass pattern	45.00% (297/660)	74.26% (75/101)	43.59% (170/390)	30.77% (52/169)	0.001 0.001 0.004 0.004

Consolidation	25.15% (166/660)	57.43% (58/101)	19.23% (75/390)	19.53% (33/169)	0.001 0.001 0.935 0.001

**Echocardiogram (cEcho)**	18.54% (266)	24.43% (64)	18.93% (81)	16.24% (121)	0.013 0.085 0.241 0.003

Left ventricular ejection fraction (LVEF) (*n* = 266)	56.89 (SD 11.89)	57.40 (SD 9.78)	54.48 (SD 13.97)	58.23 (SD 11.22)	0.008 0.421 0.083 1.000

Right ventricular fractional area (FaRV) (*n* = 166)	38.17 (SD 10.61)	34.71 (SD 7.87)	30.33 (SD 6.80)	40.20 (SD 11.41)	0.002 1.000 0.311 0.004

Left ventricular global longitudinal strain (GLS LV) (*n* = 57)	–17.31 (SD 5.18)	–13 (SD 0)	–11.2 (SD 3.65)	–18.74 (SD 4.47)	0.001 1.000 0.001 0.591

Right ventricular free wall strain (FWSRV) (*n* = 18)	–24.85 (SD 6.85)	–15 (SD 0)	N/A	–25.42 (SD 6.60)	0.144

Transmitral pattern					0.001
*LV impaired relaxation*	41.73% (111/266)	68.75% (44/64)	30.86% (25/81)	34.71% (42/121)	N/A
*Atrial fibrillation*	7.89% (21/266)	3.12% (2/64)	4.94% (4/81)	12.40% (15/121)	
*Normal*	42.48%(113/266)	26.56% (17/64)	51.85% (42/81)	44.63% (54/121)	
*Restrictive pattern*	2.63% (7/266)	0.00% (0)	4.94% (4/81)	2.48% (3/121)	
*Pseudoformal pattern*	5.26% (14/266)	1.56% (1/64)	7.41% (6/81)	5.79% (7/121)	

Abnormal motility	15.79% (42/266)	9.38% (6/64)	25.93% (21/81)	12.40% (15/121)	0.010 0.011 0.014 0.538

Pericardial effusion	10.90% (29/266)	3.12% (2/64)	4.94% (4/81)	19.01% (23/121)	0.001 0.586 0.004 0.003

**Lung ultrasound (LUS)**	7.25% (104)	22.14% (58)	0.23% (1)	6.04% (45)	0.001 0.001 0.001 0.001

Interstitial syndrome	55.77% (58/104)	96.55% (56/58)	100.00% (1/1)	2.22% (1/45)	0.001 0.850 0.001 0.001

Consolidation	10.57% (11/104)	18.96% (11/58)	0.00% (0)	0.00% (0)	0.426 N/A

Pleural effusion	9.62% (10/104)	5.17% (3/58)	0.00% (0)	15.56% (7/45)	0.197 N/A


*Notes*: * Each cell contains four p-values. The first p-value corresponds to chi-squared test (for categorical variables) or one-way ANOVA test (for numerical variables). Only a *p* ≤ 0.016 was considered statistically significance. The second, third, and fourth corresponds to multiple comparisons between groups: Mx and CAC, CAC and SAm, and SAm and Mx, respectively. We used multiple chi-squared tests and ANOVA with Bonferroni correction.

**Figure 3:**
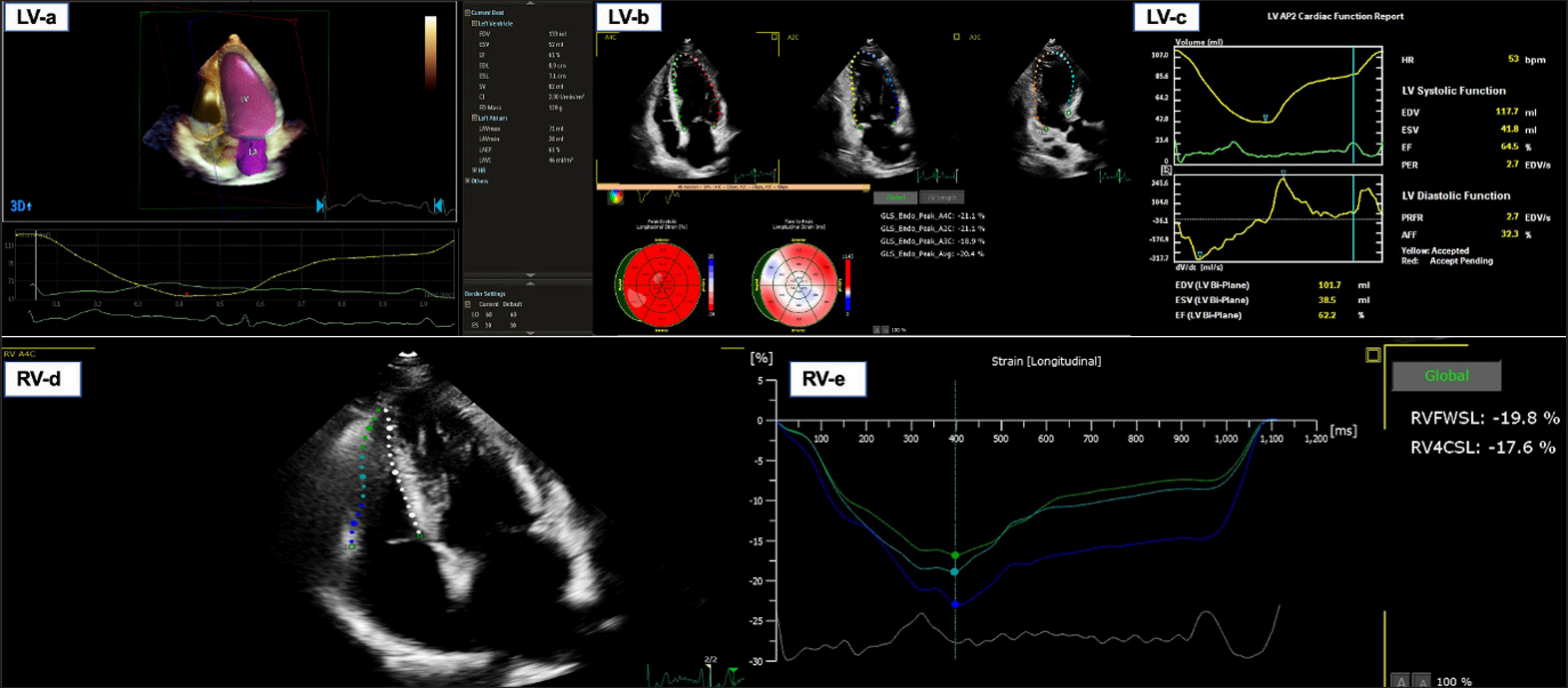
Left ventricle and right ventricle echocardiographic systolic analysis.

The right ventricular fractional area (FaRV) was performed in almost 62% of the cEcho (166 patients) with a mean value of 38% and no differences between regions. Strain echocardiography was performed only in Mx and SAm. The left ventricular global longitudinal strain (GLSLV) was performed in 21% of cEcho (57 patients) with a median of –17 (from –26 to –7) and lower in Mx (–13). The right ventricular free wall strain (FWSRV) was achieved in 7% of cEcho (18 patients) with a median value of –25 (from –40 to –7); it was also lower in Mx (–15) (Figure 3). The transmitral/tissue Doppler patterns most frequently were majority normal or impaired relaxation (42% each). The development of abnormal regional wall motility was predominant in CAC. Pericardial effusion was found in 11% of the patients, with the majority in SAm. The most frequent patterns in LUS were the interstitial syndrome (predominant in Mx and CAC) and consolidation (predominant in Mx). Logistic regression showed associations of imaging with comorbidities, complications, and evolution with an estimation of crude OR and their 95%CI.

The most frequently finding in ECG was an arrhythmia, especially in SAm.

### Associated factors related to pathological findings in images

On cX-ray, peripheral infiltrates were associated with mechanical ventilation (OR 1.66; 95%CI 1.19–2.32), mortality (OR 1.87; 95%CI 1.30–2.70) and overweight/obesity (OR 2.93; 95%CI 2.10–4.08). Meanwhile basal infiltrates were associated with mechanical ventilation (OR 2.84; 95%CI 2.05–3.94), mortality (OR 2.84; 95%CI 1.99–4.06), heart failure (OR 2.06; 95%CI 1.35–3.14), and overweight/obesity (OR 1.75; 95%CI 1.27–2.41).

On cCT, ground glass infiltrates >50% were associated with mechanical ventilation (OR 4.99; 95%CI 3.66–6.81), mortality (OR 3.68; 95%CI 2.63–5.15), heart failure (OR 2.75; 95%CI 1.73–4.36), and overweight/obesity (OR 1.84; 95%CI 1.36–2.49).

Regarding echocardiograms, the abnormal LVEF was associated with ischemic heart disease (OR 15.64; 95%CI 6.77–36.14) and the presence of heart failure (OR 7.26; 95%CI 3.73–14.16).

On LUS, interstitial syndrome resulted associated with mortality (OR 2.64; 95% 1.14–6.09).

### Other analyses

Renal failure (20%) was the most prevalent complication, with a significant predominance in Mx and SAm regions. Heart failure developed in 13% (191 patients) of the cohort, with a predominance in Mx and CAC; left ventricular failure occurred in 46%, right ventricular failure occurred in 20%, and biventricular failure occurred in 34% of cases. Lung thromboembolism was significantly higher in Mx, and acute myocardial infarction was predominant in CAC (Table 4).

**Table 4:** Complications and evolution.


	GLOBAL (*n* = 1,435)	MEXICO (MX) (*n* = 262)	CENTRAL AMERICA AND CARIBBEAN (CAC) (*n* = 428)	SOUTH AMERICA (SAM) (*n* = 745)	p-VALUE*

**Complications**					

Renal failure	20.14% (289)	22.52% (59)	13.32% (57)	23.22% (173)	0.001 0.002 0.001 0.816

Heart failure RV LV RV + LV	13.13% (191) 20.41% (39/191) 45.54% (87/191) 34.05% (65/191)	16.79% (44)	19.86% (85)	8.32% (62)	0.001 0.316 0.001 0.001

Cardiac arrest	4.81% (69)	0.76% (2)	9.58% (41)	3.49% (26)	0.001 0.001 0.001 0.021

Myocardial infarction	4.04% (58)	2.29% (6)	7.94% (34)	2.42% (18)	0.001 0.002 0.001 0.908

Myocarditis	3.41% (49)	3.44% (9)	7.71% (33)	0.94% (7)	0.001 0.023 0.001 0.005

Lung thromboembolism	3.34% (48)	10.69% (28)	1.40% (6)	1.88% (14)	0.001 0.001 0.543 0.001

Deep venous thrombosis	0.56% (8)	0.38% (1)	0.70% (3)	0.54% (4)	0.856 N/A

Takotsubo	0.21% (3)	0.38% (1)	0.23% (1)	0.13% (1)	0.746 N/A

**Evolution**					

Ward admission	49.34% (708)	61.07% (160)	47.90 (205)	46.04% (343)	0.001 0.001 0.539 0.001

ICU admission	50.66% (727)	38.93% (102)	52.10% (223)	53.96% (402)	0.001 0.001 0.539 0.001

ICU during hospitalization	58.26% (836)	60.69% (159)	57.48% (246)	57.85% (431)	0.672 N/A

Hospitalization days	17.49 (SD 17.24)	16.01 (SD 261)	15.39 (SD16.04)	19.21 (SD 19.02)	0.001 1.000 0.001 0.028

ICU days	13.56 (SD 14.2)	13.35 (SD12.68)	7.94 (SD 8.36)	16.73 (SD 16.09)	0.001 0.003 0.001 0.079

Mechanical ventilation, total patients	37.91% (544)	51.53% (135)	29.91% (128)	37.72% (281)	0.001 0.001 0.007 0.001

Mechanical ventilation, ICU patients	65.07% (544/836)	84.91% (135/159)	52.03% (128/246)	65.20% (281/431)	0.001 0.001 0.001 0.001

Mechanical ventilation duration (hours)	356.61 (SD 298.12)	308.26 (SD 271.15)	241.89 (SD 198.75)	432.11 (SD 326.17)	0.001 0.185 0.001 0.001

Mechanical ventilation duration (days)	14.85 (SD 12.42)	12.84 (SD 11.29)	10.07 (SD 8.28)	18.01 (SD 13.59)	0.001 0.185 0.001 0.001

Prone/total patients	22.37% (321)	41.98% (110)	7.24% (31)	24.16% (180)	0.001 0.001 0.007 0.001

Prone, ICU patients	37.80% (316/836)	67.30% (107/159)	11.79% (29/246)	41.76% (180/431)	0.001 0.001 0.001 0.001

Prone, mechanical ventilation	50.18% (273/544)	72.59% (98/135)	18.75% (24/128)	53.74% (151/281)	0.001 0.001 0.001 0.001

Prone duration (hours)	137.09 (SD 94.74)	141.6 (SD 91.19)	61.93 (SD 56.71)	147.28 (SD 96.68)	0.004 0.001 0.001 1.000

CPAP, total patients	13.17% (189)	12.21% (32)	10.28% (44)	15.17% (113)	0.051 N/A

CPAP, mechanical ventilation	17.83% (97/544)	9.63% (13/135)	13.28% (17/128)	23.84% (67/281)	0.001

CPAP duration (hours)	120.42 (SD 120.63)	98.25 (SD 93.32)	83.45 (SD 96.48)	141.10 (SD 131.65)	0.012 1.000 0.020 0.218

Nasal cannula	49.69% (713)	66.41% (174)	49.07% (210)	44.16% (329)	0.001 0.001 0.105 0.001

Nasal cannula duration (hours)	150.90 (SD 149.49)	214.20 (SD 161.48)	80.11 (SD 76.38)	162.60 (SD 159.90)	0.001 0.001 0.001 0.001

ECMO	0.56% (8)	0.38% (1)	0.00% (0)	0.94% (7)	0.105 N/A

ECMO (hours)	237 (SD 208.61)	504 (SD N/A)	–	198.85 (SD 192.85)	0.189 N/A

Mortality/total patients	27.60% (396)	37.40% (98)	24.07% (103)	26.17% (195)	0.001 0.001 0.424 0.001

Mortality/mechanical ventilation	56.07% (305/544)	57.78% (78/135)	59.38% (76/128)	53.74% (151/281)	0.510 N/A

Mortality/ICU patients	39.83% (333/836)	52.20% (83/159)	36.18% (89/246)	37.35%(161/431)	0.002 0.001 0.760 0.001


*Notes*: * Each cell contains four p-values. The first p-value corresponds to chi-squared test (for categorical variables) or one-way ANOVA test (for numerical variables). Only a *p* ≤ 0.016 was considered statistically significance. The second, third, and fourth corresponds to multiple comparisons between groups: Mx and CAC, CAC and SAm, and SAm and Mx, respectively. We used multiple chi-squared tests and ANOVA with Bonferroni correction. RV: right ventricular; LV: left ventricular; ICU: intensive care unit; prone: pronation; CPAP: continuous positive airway pressure; ECMO: extracorporeal membrane oxygenation.

Modalities of respiratory support had significant differences across regions: Overall, mechanical ventilation (MV) and pronation predominated in Mx ICU patients at 85% and 67%, respectively, although the mechanical ventilation duration (hours and days) was higher in SAm. CPAP in mechanical ventilated patients was more common in SAm; ECMO was rarely employed (Table 4).

The overall in-hospital mortality was 28% (95%CI: 25.2–29.9) and was significantly higher in those aged ≥65 years with no differences between gender; this is nearly similar to that published in patients from the United Kingdom [19] and slightly higher than that of Iran [20]. The higher regional mortality was in Mx (37%) with no significant differences between CAC and SAm. Rather, if we analyze for each country, then Perú and Argentina had the highest mortality with 40% and 39%, respectively. The ICU mortality was 40% and was significantly higher in México (52%). Mortality in patients with mechanical ventilation was 56% with no differences between regions (Table 4). The risk factors associated with high mortality were HBP, DBT, and overweight/obesity, as well as hospitalization location and the requirement of MV.

## Discussion

### Key results/Interpretation

The SARS-CoV-2 pandemic has had a global influence, but little is known about the effect on LATAM. The respiratory system is dramatically impacted by COVID-19, and this explains the necessary use of cX-ray and cCT overshadowing the use of cEcho and LUS. In this multicenter study, we describe the different modalities of imaging used and their findings in the management of patients affected during the early stages of the COVID-19 disease in LATAM. The three regions had expected as well as unexpected results.

The diagnosis and follow-up of pulmonary involvement was carried out using lung imaging (cX-ray or cCT). The procedures optimized the technical and human resources available in light of the personnel protection measures. Only in doubtful or complex cases were both techniques used in the same patient (18%). This could partly explain the difference in the use of these modalities between the three regions. The findings of basal and peripheral infiltrates on cX-ray and of ground glass infiltrates (>50%) in cCT were correlated with the presence of overweight/obesity, greater occurrence of heart failure, need for mechanical ventilation, and higher mortality.

According to SISIAC’s recommendations, the use of cEcho during the COVID-19 pandemic in 2020 was limited to patients with hemodynamic instability, new heart failure or ischemic heart disease, complex arrhythmias, or a high suspicion of endocarditis associated with coronavirus. The higher prevalence of heart failure, myocardial infarction, and pulmonary thromboembolism in Mx and CAC could probably explain the slightly higher use of cEcho in these regions. The development of a reduced ejection fraction during hospitalization was correlated with the presence of heart failure and ischemic heart disease.

Uncertainties about its usefulness and the lack of practice of LUS in patients with pulmonary and cardiac pathology in most LATAM countries contributed to the limit use of this technique except in Mexico and Brazil. Of note, published studies [21] that used LUS in COVID-19 patients were oriented to usefulness and results, and it remains unclear what the real rate of LUS use was. Interstitial infiltrates were correlated with higher mortality.

### Regarding regions

We hypothesize that the higher mortality in Mx could be explained by a combination of these findings: a higher prevalence of cX-ray infiltrates; cCT infiltrates >50% in more than two-thirds of the subjects; high incidence of interstitial syndrome in LUS; highest ICU stay in days; highest proportion of total patients admitted to the ICU with mechanical ventilation and pronation use (more severe ICU patients); and a higher prevalence of pulmonary thromboembolism, heart failure, and renal failure. Furthermore, the high incidence of comorbidities such as obesity, diabetes, and tobacco use suggest a high-risk cohort.

The CAC and SAm regions had fewer numbers of complications; there was less prevalence of abnormal cX-ray findings and a higher rate of moderate cCT infiltrates (25–50%). These regions had a lower proportion of total patients admitted to the ICU with mechanical ventilation and pronation use (less severe ICU patients); these could probably identify a moderate risk cohort. Interestingly, we found that the CAC region had the highest rate of hypertension and the highest proportion of regional wall motion abnormalities on cEcho, and SAm had the highest rate of arrhythmias as well as the highest prevalence of pericardial effusions. We hypothesize that the combination of these factors could probably explain the similar mortality between both regions (24% and 26%, respectively).

### Limitations/generalizability

The cohort might not be representative of all COVID-19 hospitalizations in each country because we included only patients who had some imaging performed.

Modality of choice of the images used in each patient was based on the treating physician, with considerable risk of confounding by indication: patients had certain modalities based on clinical presentation, availability of diagnostic images, and local guidelines in place for imaging. So the generalizability of the findings is limited to hospitalized COVID patients within Latin America.

Another limitation to mention is that each center had its own imaging protocols and reports, although we collected all images in a core lab and standardized its interpretation for the final analysis.

We could also consider an underreporting of ECG due to a lack of digitization.

In addition, data were collected during the first pandemic wave, and therefore findings may not reflect changes that may have occurred later.

## Conclusion

Patients hospitalized with COVID-19 had differences in the images used in the three LATAM regions. These could be explained by clinical needs, personnel protection measures, and/or hospitalization location. cCT and cX-ray were the most frequently performed modalities, and cEcho was employed only in special clinical situations.

The cardiopulmonary images used and their abnormal findings had a great impact on diagnosis and prognosis, the use of mechanical ventilation, the necessary pronation, and overall mortality. The addition of comorbidities and complications could explain the different severity rates in these patients.

## Abbreviations

BMI: Body Mass Index

CAC: Central America and Caribbean region

cCT: chest computed tomography

cEcho: echocardiogram

cMRI: cardiac magnetic resonance imaging

COPD: chronic obstructive pulmonary disease

cX-ray: chest X-ray

DBT: diabetes

ECG: electrocardiogram

FaRV: right ventricular fractional area

FWSRV: right ventricular free wall strain

GLSLV: left ventricular global longitudinal strain

HBP: high blood pressure

ICU: intensive care unit

IRB: Institutional Review Board

ISR: imaging services resources

LATAM: Latin America

LUS: lung ultrasound

LVEF: left ventricular ejection fraction

MV: mechanical ventilation

Mx: Mexican region

SAm: South America region

## Strengths

To the best of our knowledge, this is the first international, multicenter study in LATAM to characterize cardiopulmonary imaging use and their findings in patients hospitalized with COVID-19.

## Future Directions

Subsequently, imaging societies may consider developing risk scoring protocols based on the findings of the images performed.

## References

[1] Peng Z, Xing-Lou Y, Xian-Guang W, et al. A pneumonia outbreak associated with a new coronavirus of probable bat origin. Nature. 2020; 579(7798): 270–273. DOI: 10.1038/s41586-020-2012-7 32015507 PMC7095418

[2] Nanshan C, Min Z, Xuan D, et al. Epidemiological and clinical characteristics of 99 cases of 2019 novel coronavirus pneumonia in Wuhan, China: A descriptive study. Lancet. 2020; 395(10223): 507–513. DOI: 10.1016/S0140-6736(20)30211-7 32007143 PMC7135076

[3] Chaolin H, Yeming W, Xingwang L, et al. Clinical features of patients infected with 2019 novel coronavirus in Wuhan, China. Lancet. 2020; 395(10223): 497–506. DOI: 10.1016/S0140-6736(20)30183-5 31986264 PMC7159299

[4] Wu Z, McGoogan JM. Characteristics of and important lessons from the coronavirus disease 2019 (COVID-19) outbreak in China: Summary of a report of 72,314 cases from the Chinese Center for Disease Control and Prevention. JAMA. 2020; 323(13): 1239–1242. DOI: 10.1001/jama.2020.2648 32091533

[5] Fang L, Karakiulakis G, Roth M. Are patients with hypertension and diabetes mellitus at increased risk for COVID-19 infection? Lancet Respir Med. 2020; 8(4): e21. DOI: 10.1016/S2213-2600(20)30116-8 32171062 PMC7118626

[6] Richardson S, Hirsch JS, Narasimhan M, et al. Presenting characteristics, comorbidities, and outcomes among 5,700 patients hospitalized with COVID-19 in the New York City area. JAMA. 2020; 323(20): 2052–2059. DOI: 10.1001/jama.2020.6775 32320003 PMC7177629

[7] Wang D, Hu B, Hu C, et al. Clinical characteristics of 138 hospitalized patients with 2019 novel coronavirus-infected pneumonia in Wuhan, China. JAMA. 2020; 323(11): 1061–1069. DOI: 10.1001/jama.2020.1585 32031570 PMC7042881

[8] Mehraeen E, Seyed Alinaghi SA, Nowroozi A, et al. A systematic review of ECG findings in patients with COVID-19. Indian Heart Journal. 2020; 72(6): 500–507. DOI: 10.1016/j.ihj.2020.11.007 33357637 PMC7661958

[9] Moussa S, Gabriels J, Chang D, et al. Effects of chloroquine, hydroxychloroquine, and azithromycin on QTc of COVID-19 patients. Circulation Arrhythmia and Electrophysiology. 2020; 13(6): 496–504. DOI: 10.1161/CIRCEP.120.008662 PMC7299095 32347743

[10] Ming-Yen N, Elaine YPL, Jin Y, et al. Imaging profile of the COVID-19 infection: Radiologic findings and literature review. Radiology Cardiothoracic Imaging. 2020; 2(1): 1–9. DOI: 10.1148/ryct.2020200034 PMC7233595 33778547

[11] Kirkpatrick JN, Mitchell C, Taub C, et al. ASE statement on protection of patients and echocardiography service providers during the 2019 novel coronavirus outbreak: Endorsed by the American College of Cardiology. Journal American Society of Echocardiography. 2020; 33(6): 648–653. DOI: 10.1016/j.echo.2020.04.001 PMC7129086 32503700

[12] Szekely Y, Lichter Y, Taieb P, et al. The spectrum of cardiac manifestations in coronavirus disease 2019 (COVID-19)—A systematic echocardiographic study. Circulation. 2020; 142(4): 342–353. DOI: 10.1161/CIRCULATIONAHA.120.047971 32469253 PMC7382541

[13] García Fernández MA. La pandemia Covid19 y el uso de la ecocardiografía. RETIC 2020; 3(2): 1–4. DOI: 10.37615/retic.v3n2a2

[14] Li Y, Li H, Zhu S, et al. Prognostic value of right ventricular longitudinal strain in patients with COVID-19. JACC: Cardiovascular Imaging. 2020; 13(11): 2287–2299. DOI: 10.1016/j.jcmg.2020.04.014 32654963 PMC7195441

[15] Hirschaut E, Delgado C. Ecografía pulmonar: Un nuevo abordaje para cardiólogos. RETIC. 2018; 1(2): 1–7. DOI: 10.37615/retic.v1n2a2

[16] Poggiali E, Dacrema A, Bastoni D, et al. Can lung US help critical care clinicians in the early diagnosis of novel coronavirus (COVID-19) pneumonia? Radiology. 2020; 295(3): e6. DOI: 10.1148/radiol.2020200847 32167853 PMC7233381

[17] Feng P, Chuansheng Z, Tianhe Y, et al. Different computed tomography patterns of coronavirus disease 2019 (COVID-19) between survivors and non-survivors. Scientific Reports. 2020; 10(1): 11336. DOI: 10.1038/s41598-020-68057-4 32647307 PMC7347874

[18] Long C, Xu H, Shen Q, et al. Diagnosis of the coronavirus disease (COVID-19): rRT-PCR or CT? European Journal of Radiology. 2020; 126: 108961. DOI: 10.1016/j.ejrad.2020.108961 32229322 PMC7102545

[19] Docherty AB, Harrison EM, Green CA, et al. Features of 20,133 UK patients in hospital with COVID-19 using the ISARIC WHO Clinical Characterization Protocol: Prospective observational cohort study. BMJ. 2020; 369: m1985. DOI: 10.1136/bmj.m1985 32444460 PMC7243036

[20] Jalili M, Payandemehr P, Saghaei A, et al. Characteristics and mortality of hospitalized patients with COVID-19 in Iran: A national retrospective cohort study. Ann Intern Med. 2021; 174(1): 125–127. DOI: 10.7326/M20-2911 32687717 PMC7393802

[21] Volpicelli G, Gargani L, Perlini S, et al. Lung ultrasound for the early diagnosis of COVID-19 pneumonia: An international multicenter study. Intensive Care Med. 2021; 47(4): 444–454. DOI: 10.1007/s00134-021-06373-7 33743018 PMC7980130

